# 2493: GABA-A receptor binding is abnormal in sensory-motor integration brain regions in Cervical Dystonia

**DOI:** 10.1017/cts.2017.236

**Published:** 2018-05-10

**Authors:** Brian Berman, Erika Shelton, Yubin Miao

**Affiliations:** University of Colorado at Denver, Denver, CO, USA

## Abstract

OBJECTIVES/SPECIFIC AIMS: Determine whether GABA-A receptor binding is abnormal and linked to dystonia symptoms in cervical dystonia (CD). METHODS/STUDY POPULATION: There is increasing evidence that a key pathophysiological mechanism in adult-onset focal dystonia is a reduction in inhibitory control over the sensorimotor network. Results from a recent 11C-flumazenil PET imaging study suggest that abnormal inhibitory signaling in genetic and sporadic forms of dystonia may be due to reduced GABA-A binding. It remains unknown whether CD, the most common form of adult-onset focal dystonia, is associated with abnormal GABA-A binding. The goal of this research is to determine if GABA-A receptor binding is abnormal and linked to dystonia symptoms in CD. RESULTS/ANTICIPATED RESULTS: We investigated whole brain GABA-A binding in 15 CD patients (11F; 64±8 y) and 15 healthy controls (10F; 64±9 y) using 60-minute dynamic 11C-flumazenil PET scans. GABA-A receptor binding potential (BP) was estimated using a simplified reference tissue model. A 2-sample *t*-test was used to identify voxel-wise GABA-A BP differences between groups, and a regression analysis used to test for correlations between GABA-A BP and disease severity as measured with the Toronto Western Spasmodic Torticollis Rating Scale (TWSTRS). A conventional region of interest analysis was also conducted to quantify BP changes within the sensorimotor network using the automated anatomical labeling atlas. DISCUSSION/SIGNIFICANCE OF IMPACT: CD patients have reduced GABA-A receptor binding compared with healthy controls, with the greatest reduction seen within the sensorimotor region of the thalamus. Furthermore, reductions in GABA-A binding in brain regions associated with coupling sensory and motor information predict motor severity. These findings support that reduced GABAergic signaling within sensorimotor integration regions is a key mechanism underlying dystonic symptoms in CD and could help inform the development of better, more targeted treatment options.

